# Biological variation of ischaemia-modified albumin in healthy subjects

**Published:** 2008-06

**Authors:** R Govender, J De Greef, R Delport, WJH Vermaak, PJ Becker

**Affiliations:** Department of Chemical Pathology and School of Medicine, University of Pretoria, Pretoria; Department of Chemical Pathology and School of Medicine, University of Pretoria, Pretoria; Department of Chemical Pathology and School of Medicine, University of Pretoria, Pretoria; Department of Chemical Pathology and School of Medicine, University of Pretoria, Pretoria; Biostatistics Division, Medical Research Council of South Africa, Pretoria

## Abstract

**Aim:**

Ischaemia-modified albumin (IMA), as measured by the albumin-cobalt binding (ACB) test®, has been cleared by the US Food and Drug administration as a biomarker to exclude the presence of myocardial ischaemia in patients. Although there are a number of published studies detailing the clinical utility of IMA, data on the biological variation of IMA are still lacking. In this study we determined the analytical and biological variance components of ischaemia-modified albumin, and compared the distribution of IMA values in our patient population to those provided by the kit manufacturer.

**Methods:**

IMA was determined once a week for five consecutive weeks on a cohort of healthy subjects using a colorimetric method, the ACB test® on a Roche modular analyser.

**Results:**

The analytical coefficient of variation (CV_A_) was 5%, and the within-subject (CV_I_) and between-subject (CV_G_) biological variations were 3 and 7%, respectively. Analysis of the repeated measures with gender and race (black and Caucasian) as between-subject factors, and weeks (1−5) as the within-subject factor showed that gender had no significant effect on circulating IMA concentrations (*p* 5 0.3146), whereas race did have a significant effect (*p* 5 0.0062). A significant (*p* 5 0.0185) interaction was observed between gender and race.

**Conclusion:**

The ACB test® could bring a new dimension to the care and management of patients with acute coronary syndrome. Further studies for normal population distributions by gender and ethnicity, and an optimum cut-off value appear to be required.

## Summary

Each year, several million patients present to the emergency department with chest pain. According to figures from the USA, half of this group will be admitted, but only approximately 20% will actually be diagnosed with acute coronary syndrome (ACS). On the other hand, 2% of patients with acute coronary syndrome will be mistakenly discharged.^1^-^3^ As patients with ACS have a relatively higher risk of major cardiovascular events in the short term, there is considerable clinical interest and clinical research effort underway to identify biomarkers of myocardial ischaemia.^1^,^3^

A blood test that could exclude the presence of myocardial ischaemia would dramatically improve the triage process of patients with acute coronary symptoms, decrease the number of hospital admissions and reduce the overall cost of healthcare. Clinical studies have shown that ischaemia-modified albumin (IMA), as measured by the albumin-cobalt binding (ACB) test®, has been cleared by the US Food and Drug Administration as a possible early indicator of myocardial ischaemia.^4^-^7^ This test is a biochemical assay based on the observation that human albumin has the capacity to bind transition metals. In the presence of ischaemia (myocardial and elsewhere), the amino or N-terminal of albumin is modified and subsequently affects transition metal binding.^8^ This modified albumin, with a lower transition metalbinding capacity, is known as IMA.

IMA rises within minutes of the onset of myocardial ischaemia and returns to baseline within six hours of restoring perfusion.^9^ Previously published studies describe IMA as a risk-stratification tool for suspected ACS. In selected low-risk emergency department patients defined by an electrocardiogram (which is non-diagnostic for ischaemia), a negative troponin and a normal IMA test, discharge can be considered. Hence IMA is used as a ‘rule-out’ test in selected patients with ACS.^1^,^9^

Although there are a number of studies detailing the clinical utility of IMA, its biological variation is still lacking. Biological variation studies are essential prerequisites to the introduction of any new biomarker. This data should be generated early in the course of evaluation of new tests as quantitative data obtained can be used to set desirable analytical quality specifications, assess the utility of conventional reference ranges, and define the significance of changes in serial results.^10^,^11^

In this study we performed IMA testing on a cohort of healthy subjects over a five-week period to determine the analytical and the biological variance components of ischaemia-modified albumin.

## Materials and methods

The subject population consisted of 17 apparently healthy volunteers; seven men, two Caucasian, five black (age range 43−61 years, median age 50 years) and 10 women; six Caucasian, four black (age range 26−61 years, median age 41 years). All participants were required to complete a modified RAND 36-item Health Survey 1.0 questionnaire^12^,^13^ to determine their health status and were observed to be healthy. Informed consent prior to enrolment in the study was given and the Human Ethics committee of the University of Pretoria approved the study. No exclusion criteria were applied.

Blood samples were collected once a week for five consecutive weeks. To minimise pre-analytical variation, the specimens were collected at a constant time and by the same phlebotomist, and the collection technique was standardised. The samples were separated and then frozen at −70°C within one hour of collection and were analysed according to the manufacturer’s instruction.

Ischaemia-modified albumin was determined using a colorimetric method, the ACB test® on a Roche modular analyser. This test measures the cobalt-binding capacity of albumin in a serum sample. A cobalt solution is added to the serum. Cobalt not bound to the N-terminal of albumin is detected using dithiothreitol as a colorimetric indicator. In individuals with ischaemia, cobalt does not bind to the modified N-terminal of IMA, leaving more free cobalt to react with dithiothreitol.

The instrument was set up according to the manufacturer’s instructions and was calibrated before the analyses were performed. Duplicate analyses were performed on all samples in a single batch. The analytical coefficient of variation for the low control was 4.22% (range 55−71 U/ml), for the medium control, 2.01% (range 69−91 U/ml) and for the high control, 1.24% (range 102−130 U/ml).

The hierarchical design of analysis of variance was determined using Statistix 8.0 software, with the variance components then being used to calculate the analytical variation (CV_A_), within-subject variation (CV_I_) and the between-subject variation (CV_G_) according to Fraser and Harris. The analytical goals for imprecision (0.5 3 CV_I_) and bias [0.25 × (CV_I_ + CV_G_)^1/2^] were also determined.^10^,^11^ The index of individuality was calculated by

$\frac{{\left(CV_{A}^{2}+CV_{I}^{2}\right)}^{1/2}}{CV_{G}}\;as\;CV_{A}>CV_{I}$

Analyses of the measurements were performed, with gender and race (black and Caucasian) as between-subject factors and weeks (1−5) as the within-subject factor, using an appropriate ANOVA for repeated measures.

## Results

The mean, within-run analytical variation, between- and within-subject biological variation, goals for imprecision and bias, and the index of individuality are shown in Table 1. Analysis of the repeated measures with gender and race as between-subject factors, and weeks as the within-subject factor showed that gender had no significant effect on circulating IMA concentrations (*p* 5 0.3146), whereas race did have a significant effect (*p* 5 0.0062). A significant (*p* 5 0.0185) interaction was observed between gender and race. From Table 2, the relationship between gender and race is evident and it seems reasonable to ascribe the interaction to the very different outcomes for males between the races. Furthermore, the within-factor week (Table 3) was not significant (*p* 5 0.1915) and the interaction in terms of week with gender and race were omitted in this final analysis as they were insignificant in the initial analysis and were then pooled with the error term.

**Table 1.** Calculations Relating To The Biological VA Riation Of IMA

*Parameter*	
CV_A_ (%)	5.04
CV_I_ (%)	2.89
CV_G_ (%)	6.76
Index of individuality	0.86
Desirable analytical imprecision (%)	1.45
Desirable analytical bias (%)	1.84

CV_A_: analytical coefficient of variation; CV_I_: within-subject coefficient of variation; CV_G_: between-subject coefficient of variation.

**Table 2.** Race And Gender As Between-Subject Factors

	*Gender*		
*Race*	*Male*	*Female*	*Total*
Caucasian			
No of observations	10	30	40
Mean	94.95	106.63	103.71
Standard deviation	4.80	7.35	8.47
Black			
No of observations	25	20	45
Mean	113.38	108.70	111.30
Standard deviation	6.94	6.80	7.20
Total			
No of observations	35	50	85
Mean	108.11	107.46	107.73
Standard deviation	10.56	7.14	8.66

**Table 3.** IMA Concentrations With Weeks As The Within-Subject Factor

*Week*	*Number of observations*	*Mean*	*Standard deviation*
1	17	106	8.41
2	17	109.77	10.00
3	17	108.62	7.26
4	17	107.38	9.63
5	17	106.88	8.25

## Discussion

Using a standard protocol for sample collection, pre-analytical variation was considered negligible or an intrinsic component of the within-subject biological variation. As shown in previous studies, estimates of biological variation in a small group of apparently healthy subjects may be useful for a variety of purposes and these estimates should be similar across studies, in theory at least, since the results are quantitative components of homoeostatic mechanisms in a single animal species.^10^,^11^,^14^ It is widely accepted that the best strategy for determining the standards of analytical performance (precision and bias) in order to provide optimal patient care are best derived from data on biological variation.^10^,^11^ This study therefore investigated the potential use of IMA based on these calculations.

**Fig. 1.**
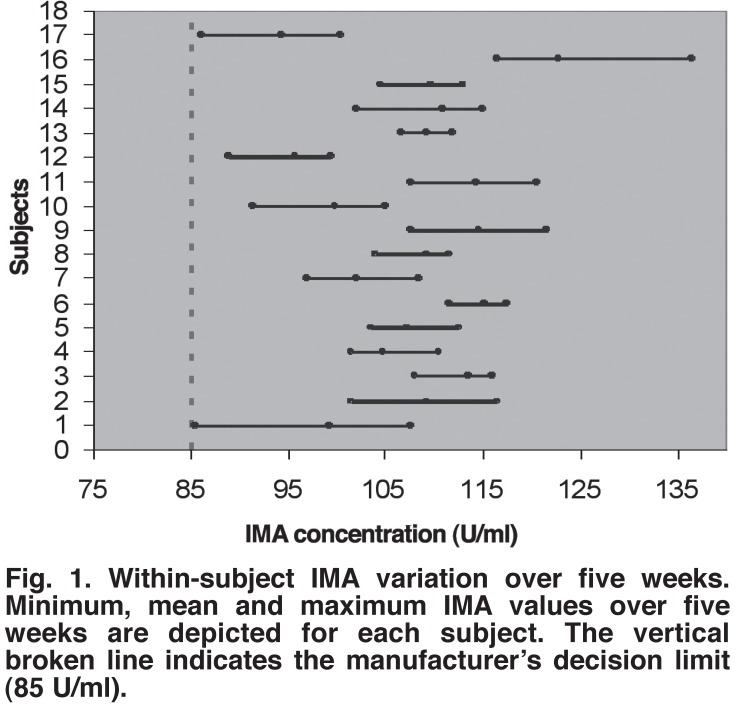
Within-subject IMA variation over five weeks. Minimum, mean and maximum IMA values over five weeks are depicted for each subject. The vertical broken line indicates the manufacturer’s decision limit (85 U/ml).

The IMA values obtained in our population were considerably higher (Fig. 1) than specified by the kit manufacturer, but similar to values obtained in other clinical studies.^2^,^15^ The sample size of this study was deemed too small to allow a valid calculation of the reference range. Laboratories are encouraged by the manufacturer to establish their own optimal IMA clinical cut-off concentrations as the values may vary, depending on geographic, patient, dietary and environmental factors.^16^ In this study, these non-random variations were not accounted for. Furthermore, serum albumin, which could affect IMA results when using the ACB test, was not measured in this study population. The main reason for this omission was the supposition that the population comprised ‘apparently healthy’ individuals with normal serum albumin concentrations.

Several recent studies^15^,^17^ have shown that storage of IMA samples at 4°C or −20°C have resulted in higher values compared to real-time analysis. Although the effect of storing samples at −70°C (as in this study) was not assessed by means of a stability study, it can be inferred that the effect would be similar to that reported previously. This may explain why higher values were obtained compared with the values reported by the manufacturer. Further studies on the effect of sample storage at −70°C on IMA concentrations are indicated.

The observed precision for IMA determination (5.04%), although acceptable, shows less than desirable analytical imprecision (1.45%), which indicates that the current method for IMA assay on the Roche modular analyser may warrant further improvement and optimisation. It was difficult to decide whether the goals for bias were met, which if met, would allow the use of common reference intervals throughout a geographical area. Standardisation of the IMA assay will, however, ensure that these goals are met in the near future.

Harris has shown that when the within-subject variation is greater than the between-subject variation, conventional reference values will be of use, but when the between-subject variation is greater than the within-subject variation, reference values will be of little use for monitoring change.^18^,^19^ More formally, reference values are of marked usefulness only if the index of individuality is greater than 1.4. In this study, the index of individuality was less than 1.4, which may be explained by the interaction between race and IMA concentrations.

It can therefore be deduced that IMA determination may currently not be a good test for detecting latent or early disease, since an individual may have a value that is very unusual for him/her but still falls well within conventional population-based reference limits. Although in practice, a decision cut-off is used for the interpretation of IMA results, this marked individuality would still mean that false negative results could occur, which in theory would reduce the sensitivity of the IMA assay as an exclusion test of myocardial ischaemia. The significant interaction between race and IMA values obviously prompts further investigation.

Factors other than biological variation that may need to be considered before routinely employing the IMA test are briefly alluded to. The rapid turn-around time (dwell time of ± 20 min) makes IMA a suitable cardiac marker for the early diagnosis of myocardial ischaemia. The specificity of IMA may, however, not be optimal as IMA may be elevated in patients with active cancer, bacterial or viral infections, end-stage renal disease, liver cirrhosis, brain ischaemia, peripheral arterial disease and trauma. Since the meaning of a raised IMA value is not clearly understood, it has been suggested that such results should prompt the clinician to resume an ACS evaluation as per their usual standard.^9^

Furthermore, sample and reagent lability needs to be kept in mind. The labile nature of IMA requires that the sample be analysed within 2.5 hours of sample collection or refrigerated/frozen until analysis. The dithiothreitol reagent and hence the full kit is only stable for 14 days. The current high cost of the test may also limit widespread use.

## Conclusion

The ACB test® could bring a new dimension to the care and management of patients with acute coronary syndrome. IMA can be measured accurately, reliably and within an acceptable time period to be useful in the evaluation of the patient with ACS, however, further studies for normal population distributions by gender and ethnicity, and an optimum cut-off value are still required.
